# Supplementary material for: Development of a risk prediction model for sepsis-related delirium based on multiple machine learning approaches and an online calculator
Source: PLoS One. 2025 Jul 16;20(7):e0323831. doi: 10.1371/journal.pone.0323831 (PMC12266397; doi:10.1371/journal.pone.0323831)
Supplement: S1 Table — Gradient Boosting Machine: GBM; Support Vector Machine: SVM; Random Forest: RF; Extreme Gradient Boosting: XGBoost; Adaptive Boosting: AdaBoost; Light Gradient Boosting Machine: LightGBM. (DOCX) [file pone.0323831.s001.docx]

To optimize the hyperparameters of the machine learning models, we employed grid search and ten-fold cross-validation. Taking the hyperparameters of GBM as an example, we first optimized three hyperparameters: 'n.trees', 'interaction.depth', and 'shrinkage'. For 'n.trees', an excessive number of trees may lead to overfitting, so to find a balance between model performance and computational cost, we set the candidate values in the range of 100 to 110. For 'interaction.depth', to control the complexity of the trees and the capacity of the model, we defined the candidate values in the range of 4 to 5. For 'shrinkage', which determines the contribution of each tree to the overall model, we set the candidate values in the range of 0.05 to 0.1. Subsequently, we used ten-fold cross-validation to evaluate the performance of each set of candidate values on the training set, in order to identify the optimal combination of hyperparameters. The results of hyperparameter optimization for GBM, SVM, Neural Network, RF, XGBoost, AdaBoost, LightGBM, and Logistic models are presented in S1 Table.

| **Model** | **Hyperparameter** | **Tuning scope** | **Optimal hyperparameter** |
| --- | --- | --- | --- |
| GBM | n.trees | (100, 110) | 110 |
|  | interaction.depth | (4, 5) | 5 |
|  | shrinkage | (0.05, 0.1) | 0.06 |
| SVM | sigma | (0.01,0.001) | 0.001 |
|  | C | (0.1,0.2) | 0.1 |
| NeuralNetwork | size | (3, 6) | 6 |
|  | decay | (0.5, 0.6) | 0.6 |
| RF | mtry | (9, 11) | 11 |
| Xgboost | nrounds | (4, 5) | 5 |
|  | max_depth | (2, 3) | 3 |
|  | eta | (0.01, 0.001) | 0.001 |
|  | gamma | (0.1, 0.5) | 0.5 |
| Adaboost | mfinal | (1, 2) | 2 |
|  | maxdepth | (2, 3) | 2 |
| LightGBM | min_data | (1, 5) | 1 |
|  | learning_rate | (0.1, 1) | 1 |
|  | num_threads | (1, 10) | 2 |

S1 Table Hyperparameter settings for eight models. Gradient Boosting Machine: GBM; Support Vector Machine: SVM; Random Forest: RF; Extreme Gradient Boosting: XGBoost; Adaptive Boosting: AdaBoost; Light Gradient Boosting Machine: LightGBM.
