# Supplementary material for: Development of a risk prediction model for sepsis-related delirium based on multiple machine learning approaches and an online calculator
Source: PLoS One. 2025 Jul 16;20(7):e0323831. doi: 10.1371/journal.pone.0323831 (PMC12266397; doi:10.1371/journal.pone.0323831)
Supplement: S2 Table — (DOCX) [file pone.0323831.s002.docx]

| **The example parameters** | |
| --- | --- |
| AKI | YES |
| CRRT | YES |
| VP | YES |
| Midazolam | YES |
| GCS | 8 |
| Age | 88 |
| ICU_Day | 10 |
| Chloride | 75 |
| SOFA | 22 |
| MAP | 120 |
| Sodium | 150 |
| RDW | 29 |
| Lactate | 1 |
| Magnesium | 15 |
| PLT | 500 |
| Temperature | 36 |
| MCV | 100 |
