# Supplementary material for: Development of a risk prediction model for sepsis-related delirium based on multiple machine learning approaches and an online calculator
Source: PLoS One. 2025 Jul 16;20(7):e0323831. doi: 10.1371/journal.pone.0323831 (PMC12266397; doi:10.1371/journal.pone.0323831)
Supplement: S3 Table — (DOCX) [file pone.0323831.s003.docx]

| **Variables** | **Total (n = 3023)** | **NSAD (n = 1428)** | **SAD (n = 1595)** | **p** |
| --- | --- | --- | --- | --- |
| **Demographics** |  |  |  |  |
| Age(years) | 71 (61, 78) | 72 (62, 78) | 71 (59, 78) | 0.579 |
| Gender(%) |  |  |  | < 0.001 |
| Female | 1355 (45) | 695 (49) | 660 (41) |  |
| Male | 1668 (55) | 733 (51) | 935 (59) |  |
| **Comorbidity** |  |  |  |  |
| Hypertension(%) |  |  |  | 0.358 |
| No | 1840 (61) | 882 (62) | 958 (60) |  |
| Yes | 1183 (39) | 546 (38) | 637 (40) |  |
| AKI(%) |  |  |  | < 0.001 |
| No | 1440 (48) | 576 (40) | 864 (54) |  |
| Yes | 1583 (52) | 852 (60) | 731 (46) |  |
| T2DM(%) |  |  |  | 0.383 |
| No | 1977 (65) | 922 (65) | 1055 (66) |  |
| Yes | 1046 (35) | 506 (35) | 540 (34) |  |
| HF(%) |  |  |  | 0.622 |
| No | 1880 (62) | 881 (62) | 999 (63) |  |
| Yes | 1143 (38) | 547 (38) | 596 (37) |  |
| **ICU interventions** |  |  |  |  |
| MV(%) |  |  |  | < 0.001 |
| No | 1938 (64) | 1049 (73) | 889 (56) |  |
| Yes | 1085 (36) | 379 (27) | 706 (44) |  |
| CRRT(%) |  |  |  | 0.639 |
| No | 2885 (95) | 1366 (96) | 1519 (95) |  |
| Yes | 138 (5) | 62 (4) | 76 (5) |  |
| Midazolam(%) |  |  |  | < 0.001 |
| No | 2119 (70) | 1118 (78) | 1001 (63) |  |
| Yes | 904 (30) | 310 (22) | 594 (37) |  |
| VP(%) |  |  |  | 0.026 |
| No | 1981 (66) | 1000 (70) | 981 (62) |  |
| Yes | 1042 (34) | 428 (30) | 614 (38) |  |
| **Scoring system** |  |  |  |  |
| SOFA(score) | 4 (3, 7) | 4 (2, 6) | 5 (3, 7) | < 0.001 |
| SAPSII (score) | 38 (31, 47) | 37 (31, 45) | 39 (31, 48) | < 0.001 |
| GCS (score) | 15 (14, 15) | 15 (14, 15) | 14 (13, 15) | < 0.001 |
| **Vital signs** |  |  |  |  |
| HR(minute) | 92 (79, 107) | 93 (79, 108) | 92 (79, 107) | 0.505 |
| SBP(mmHg) | 119 (103, 138) | 118 (102, 136) | 121 (104, 139) | 0.019 |
| DBP(mmHg) | 63 (53, 76) | 62.5 (52, 75) | 64 (54, 76) | 0.145 |
| MAP(mmHg) | 76 (66, 89) | 75 (65, 89) | 77 (67, 90) | 0.043 |
| RR(minute) | 19 (16, 24) | 20 (16, 24) | 19 (16, 23) | 0.287 |
| SPO2(%) | 97 (94.5, 100) | 97 (94, 99) | 97 (95, 100) | < 0.001 |
| Temperature(°C ) | 36.72 (36.28, 37.17) | 36.78 (36.33, 37.17) | 36.72 (36.22, 37.17) | 0.064 |
| ICU_Day(days) | 3.21 (1.91, 6.84) | 2.62 (1.75, 4.34) | 4.38 (2.18, 9.13) | < 0.001 |
| **Laboratory Results** |  |  |  |  |
| RBC(10^12^/L) | 3.46 (3.02, 3.95) | 3.43 (2.96, 3.87) | 3.48 (3.07, 3.99) | < 0.001 |
| WBC(10^9^/L) | 10.2 (7, 14.7) | 9.9 (6.8, 14.6) | 10.4 (7.1, 14.9) | 0.048 |
| Platelet(10^9^/L) | 203 (138, 284) | 195 (128.75, 277) | 206 (146, 289) | < 0.001 |
| Hemoglobin(g/dL) | 10.4 (9.1, 11.8) | 10.2 (8.9, 11.5) | 10.5 (9.3, 12) | < 0.001 |
| RDW(%) | 15.2 (13.9, 16.9) | 15.2 (14, 16.9) | 15.1 (13.9, 16.8) | 0.28 |
| HCT(%) | 31.4 (27.6, 35.5) | 31 (27.1, 35) | 31.7 (28.2, 35.8) | < 0.001 |
| MCH(pg picograms) | 30.3 (28.7, 31.9) | 30.1 (28.5, 31.7) | 30.5 (29, 32.1) | < 0.001 |
| MCHC(g/dL) | 33.2 (32.1, 34.3) | 33 (32, 34.1) | 33.4 (32.3, 34.4) | < 0.001 |
| MCV(fL femtoliters) | 91 (87, 96) | 91 (86, 96) | 91 (87, 96) | 0.063 |
| INR(ratio) | 1.3 (1.1, 1.7) | 1.3 (1.2, 1.8) | 1.3 (1.1, 1.6) | < 0.001 |
| PT(seconds) | 14.5 (13, 17.6) | 14.8 (13, 18.8) | 14.3 (13, 16.65) | < 0.001 |
| APTT(seconds) | 31.2 (27.2, 38) | 32 (28, 39.08) | 30.6 (26.6, 36.8) | < 0.001 |
| Creatinine(mg/dL) | 1.1 (0.8, 1.9) | 1.3 (0.9, 2.1) | 1.1 (0.8, 1.8) | < 0.001 |
| BUN(mg/dL) | 25 (16, 43) | 27 (17, 45.25) | 23 (15, 40.5) | < 0.001 |
| Anion Gap(mEq/L) | 14 (12, 17) | 14 (12, 17) | 14 (12, 17) | 0.284 |
| PH | 7.39 (7.32, 7.44) | 7.39 (7.33, 7.44) | 7.4 (7.32, 7.44) | 0.599 |
| Bicarbonate(mEq/L) | 23 (20, 27) | 23 (20, 26) | 24 (21, 27) | 0.001 |
| Calcium(mg/dL) | 8.3 (7.7, 8.8) | 8.2 (7.6, 8.7) | 8.4 (7.8, 8.85) | < 0.001 |
| Magnesium(mg/dL) | 1.9 (1.7, 2.1) | 1.9 (1.6, 2.1) | 1.9 (1.7, 2.1) | 0.279 |
| Chloride(mEq/L) | 104 (100, 108) | 104 (99, 108) | 104 (100, 108) | 0.01 |
| Potassium(mEq/L) | 4.1 (3.7, 4.6) | 4.1 (3.7, 4.5) | 4.1 (3.7, 4.6) | 0.923 |
| Sodium(mEq/L) | 138 (135, 141) | 138 (135, 141) | 139 (136, 142) | < 0.001 |
| Lactate(mmol/L) | 1.5 (1.1, 2.3) | 1.6 (1.1, 2.3) | 1.5 (1.1, 2.2) | 0.061 |
| Glucose(mg/dL) | 123 (100, 158) | 122 (99, 158) | 124 (100, 159) | 0.488 |

**SOFA**, Sequential Org**an Failure Assessment; SAPSII, Simplified Acute Physiologic Score II; GCS, Glasgow Coma Scale; SBP, Systolic Blood Pressure; DBP, Diastolic Blood Pressure; MAP, Mean Arterial Pressure; RDW, Red Cell Distribution Width; MCHC, Mean Corpuscular Hemoglobin Concentration; MCV, Mean Corpuscular Volume; BUN, Blood Urea Nitrogen; AKI, Acute Kidney Injury; T2DM, Type 2 Diabetes Mellitus; HF, Heart Failure; MV, Mechanical Ventilation; RBC, Red Blood Cell; WBC, White Blood Cell; HCT, Hematocrit; MCH, Mean Corpuscular Hemoglobin; INR, International Normalized Ratio; PT, Prothrombin Time; APTT, Activated Partial Thromboplastin Time.**
