# Supplementary material for: Development of a risk prediction model for sepsis-related delirium based on multiple machine learning approaches and an online calculator
Source: PLoS One. 2025 Jul 16;20(7):e0323831. doi: 10.1371/journal.pone.0323831 (PMC12266397; doi:10.1371/journal.pone.0323831)
Supplement: Attachment — Submitted filename: Response to Reviewers.docx [file pone.0323831.s004.docx]

**Dear Reviewer,**

On behalf of all authors, we sincerely appreciate your thorough review of our manuscript and the valuable insights and suggestions you provided. Your thoughtful feedback has been essential in enhancing the quality and clarity of our research. We have carefully considered each of your comments and have revised the manuscript accordingly. We believe these modifications have significantly strengthened both the overall presentation and scientific rigor of our work. Thank you once again for the time and expertise you dedicated to this review. We look forward to your further evaluation of the revised manuscript. Attached please find our point-by-point responses to your recommendations, with all corresponding changes clearly highlighted in the manuscript for your reference.

1. Methods Section. Clarify SMOTE Application: State explicitly that SMOTE was applied only to the training set.

**Reply:** We sincerely appreciate your valuable comments. We fully endorse your perspective that strictly limiting the application of the SMOTE to the training set is crucial for preventing data leakage and ensuring unbiased model evaluation results. To address this, we have added clear and explicit supplementary clarification in Section "**2.5 Model development and evaluation**" of the manuscript.

**Revised Text (lines 196 - 205):**

*“We employed a random sampling method to allocate the patients included in the study into the training set and validation set in a ratio of 7:3. The data distribution in the training set was as follows:2,355 SAD cases and 8,930 Non-SAD cases. Given that class imbalance may introduce bias into the machine learning prediction model, we conducted a supplementary analysis comparing the use of oversampling, undersampling, and the Synthetic Minority Over-sampling Technique (SMOTE) to balance the training set. Critically, SMOTE was applied exclusively to the training set. After balancing using the SMOTE algorithm, the final training set comprised 7,065 SAD cases and 7,065 Non-SAD cases. This approach effectively mitigates model bias induced by class imbalance while avoiding the risk associated with oversampling.”*

2. Address RF Overfitting: Discuss pruning, feature reduction, or ensemble methods to improve generalizability.

**Reply:** Thank you for your valuable feedback. While RF theoretically mitigate overfitting risks through bagging and feature randomness, they can still exhibit overfitting in practical applications, particularly when data contains noise, sample sizes are limited, or feature dimensionality is high. For the RF model implementation in this study, we utilized the caret package in R for model training and hyperparameter tuning. Through a rigorous optimization process employing grid search and 10-fold cross-validation, we identified the optimal hyperparameter combination as mtry = 11. This configuration achieves the best balance under the current model architecture and data characteristics. Despite thorough debugging and optimization of relevant parameters, the model still demonstrates signs of overfitting. It is important to note that the RF implementation interface within the caret package imposes certain limitations on parameter adjustment. Attempts to introduce additional parameters (such as min_samples_leaf or max_depth) triggered compatibility errors, preventing us from further adjusting model complexity via traditional pruning methods. This technical constraint prompted our exploration of alternative overfitting mitigation strategies, specifically focusing on sample engineering and feature selection. To address this issue, we employed undersampling, oversampling, and the SMOTE algorithm to augment samples near the decision boundary of the minority class. This significantly increased the number of delirium cases while ensuring clinical plausibility of the data. These measures effectively alleviated overfitting risks stemming from insufficient samples and enhanced the model's generalization capability. We sincerely appreciate your insightful comments. Moving forward, we plan to systematically enhance the generalization ability of medical models in data-constrained scenarios through multidimensional strategies, including model architecture innovation, continual learning mechanisms, and deeper clinical integration.

**Revised Text (lines 437 - 446):**

*“In this study, we compared the performance of eight machine learning models. Although RF theoretically mitigate overfitting risks through bagging and random feature selection, they can still exhibit significant overfitting in practical applications, particularly with noisy datasets or limited sample sizes. To address this, we implemented rigorous optimization procedures, including grid search, 10-fold cross-validation, and the SMOTE algorithm to alleviate sample insufficiency. Nevertheless, even after applying SMOTE to augment the training dataset, the RF model continued to demonstrate a tendency toward overfitting. Consequently, based on comprehensive performance evaluation, we ultimately identified GBM as the optimal model for this study.”*

3. Reconcile Feature Count: Justify using only the 17 shared features instead of union/intersection of Boruta/Lasso/MLR.

**Reply:** Thank you for raising this valuable point. Your question regarding our feature selection strategy touches upon one of the core aspects of model development. Our decision to utilize the 17 features jointly identified by all three methods (Boruta, Lasso regression, and MLR), rather than taking their union or a simple intersection, primarily stems from the following rigorous considerations: (1) Robustness and Reduced Bias: These three feature selection methods employ distinct criteria for evaluating feature importance. A feature consistently identified as significant across all three methods demonstrates robust predictive power and stability under diverse algorithmic frameworks and evaluation metrics. This significantly mitigates the model's sensitivity to the potential bias inherent in any single feature selection approach, thereby enhancing the credibility of our results. Opting for the intersection ensures each included feature has undergone stringent multi-method validation. This substantially strengthens the clinical plausibility of the feature set and the model's interpretability – both paramount for clinical risk prediction models. (2) Clinical Usability and Pragmatism: A key objective of this study is to develop an online risk calculator for clinicians to perform rapid patient risk assessments in settings like the bedside or emergency department. The practical constraints of clinical application necessitate a model with inputs that are concise, efficient, and readily obtainable. Utilizing the union of features would significantly increase data collection time and cost, undermining clinician adoption and feasibility. In contrast, the set of 17 shared features strikes the optimal balance: it preserves core predictive performance (Internal Validation AUC: 0.73; External Validation AUC: 0.70) while perfectly aligning with our goal of building a highly robust, interpretable, and clinically usable model and calculator. It maximizes feature economy without compromising the predictive efficacy required for clinical needs, effectively mitigates overfitting risk, and ultimately serves the practical clinical deployment and dissemination of the online calculator.

**Revised Text (lines 190 - 194):**

*“To ensure maximum robustness of the selected features, mitigate overfitting risks, enhance clinical interpretability, and meet the requirements for both feature quantity and practicality in subsequent online calculators, this study ultimately selected the intersecting features identified by Boruta, Lasso, and MLR as the modeling feature set.”*

**Revised Text (lines 428 - 432):**

*“The cross-verified features identified through these three complementary methods deliver optimal predictive power and generalizability. This strategy ensures model robustness and parsimony, simultaneously providing an operationally efficient, computationally feasible, and clinically actionable feature set for online calculator implementation.”*

4. Resolve Metric Conflicts: Recheck AdaBoost validation calculations; consider data leakage or class imbalance in validation.

**Reply:** Thank you for raising these valuable points. Your concerns regarding the reliability of the AdaBoost model validation results, and the potential impact of data leakage or class imbalance, are critically important. We have conducted a rigorous and meticulous review and analysis of the issues you raised, in accordance with your suggestions. We took seriously your concerns about potential conflicts or computational errors in the AdaBoost model's results on the validation set. To ensure the accuracy and reproducibility of our findings, we immediately re-executed the entire prediction and evaluation process for the AdaBoost model on the original validation set. We re-ran the complete code for building the AdaBoost model and generating predictions on the validation set, reconfirming that the validation samples, feature data, and labels were identical to the original partition. After strict re-calculation and verification, we confirmed that the performance metrics reported in the paper for the AdaBoost model on the validation set are accurate. We fully understand the significant impact data leakage can have on the validity of model evaluation. Furthermore, we completely agree that SAD, as a relatively rare complication, presents class imbalance in the dataset. We wish to clarify that oversampling, undersampling, and SMOTE techniques were applied exclusively to the training set during model development to balance the positive and negative class sample ratios. This was done to enhance the model's learning capability for the SAD sample group. Crucially, performance evaluation on the validation and test sets was conducted using the original, unaltered data distribution. This ensures that the evaluation results reflect the model's generalization performance on real-world, imbalanced data. By applying SMOTE to the training set, the AdaBoost model achieved an AUC value of 0.69 on the internal validation set, representing a significant improvement. Throughout the entire research process, we strictly adhered to best practices to prevent data leakage. Sampling techniques were confined solely to the training set for model development, thereby safeguarding the reliability of the model performance evaluation. Thank you for your insightful observations. This process of verification and reflection has significantly enhanced the rigor of our study.

5. Visualize Calculator: Add a screenshot of the web tool with example inputs/outputs (Fig 7A).

**Reply:** We sincerely appreciate your interest in the "Online Calculator for Sepsis-Associated Delelirium Risk" developed in our study and thank you for your valuable suggestions. We fully agree that clearly demonstrating the calculator's operational interface within the manuscript—particularly by including concrete input examples alongside corresponding predictive outputs—is essential for readers to intuitively understand the tool's application workflow, functionality, and real-world translation of the model. In accordance with your recommendation, we have redesigned Figure 7 to present a screenshot of the recalculator's web interface redeployed using our final optimized model. To ensure transparency and reproducibility, we have provided the complete set of sample input data used to generate the Figure 7 screenshot in S2 Table. This table details the specific values entered for each feature variable and their corresponding predictive outputs, enabling reviewers and readers to independently verify the screenshot contents or perform simulated operations. We are confident that incorporating this interface visualization with authentic input-output examples will significantly enhance the manuscript's readability, credibility, and practical value to its audience. Thank you once again for this highly constructive feedback, which has substantially strengthened our presentation.

**Revised Text (lines 382):**

*“The example parameters can be found in S2 Table.”*

S2 Table **Example input parameters for the online risk calculator predicting SAD.**

| **The example parameters** | |
| --- | --- |
| AKI | YES |
| CRRT | YES |
| VP | YES |
| Midazolam | YES |
| GCS | 8 |
| Age | 88 |
| ICU_Day | 10 |
| Chloride | 75 |
| SOFA | 22 |
| MAP | 120 |
| Sodium | 150 |
| RDW | 29 |
| Lactate | 1 |
| Magnesium | 15 |
| PLT | 500 |
| Temperature | 36 |
| MCV | 100 |

**Fig 7 Online web calculator for predicting the probability of SAD occurrence.**

6. Compare GBM performance against published SAD models (e.g., AUCs, features used).

**Reply:** We sincerely appreciate the your valuable feedback. In response to the request for comparative analysis of our GBM model's performance metrics (e.g., AUC) and feature sets against published SAD models, we have systematically reviewed high-impact SAD prediction studies in recent literature and conducted a multidimensional comparison between our GBM model and key benchmark models. Furthermore, unlike studies that solely publish prediction formulas, we have developed a web-based SAD risk calculator to enhance accessibility for clinical practitioners. We reiterate our gratitude for the reviewers’ insightful comments, which have significantly enhanced the completeness of our research.

**Revised Text (lines 447 - 463):**

*“However, in existing research, Zhang Y et al. utilized Lasso regression to identify key predictors for SAD patients and subsequently constructed an XGBoost prediction model based on these factors. Their study ultimately identified lower GCS scores, sedative medication use, and concomitant AKI as risk factors for SAD development, findings consistent with the results of the present study. This model demonstrated robust predictive performance on both the internal validation set (AUC = 0.793) and the external validation set (AUC = 0.701). By contrast, the AUC value of the model developed in the current study is comparatively lower. This discrepancy may stem from differences in data preprocessing pipelines and the specific feature sets incorporated. Notably, Zhang et al.'s model did not include core variables such as lactate levels and ICU_Day. The omission of such critical information may have limited its predictive capability (33). On the other hand, Gu Q et al. employed MLR to identify higher SOFA scores, elevated lactate levels, elevated phosphate levels, and MV use as independent risk factors for SAD. They subsequently developed a nomogram for risk prediction based on these factors. However, unlike the present study, their model lacks an external validation cohort. Consequently, its generalizability remains unassessed, posing challenges for real-world clinical application and implementation (34).”*

7. Discuss clinical implications of nonlinear relationships (e.g., GCS 1–3 vs. 15).

**Reply:** Thank you for your deep interest in model interpretability. Our SHAP dependence plots revealed a non-linear relationship between GCS scores and delirium risk, specifically showing that both the lowest GCS values (1-3) and the highest GCS values (14-15) contributed significantly less to the predicted risk. We have elaborated on the important underlying clinical mechanisms behind this finding in the Discussion section. We greatly appreciate your valuable suggestions, which prompted us to delve deeper into the clinical significance of this non-linear relationship.

**Revised Text (lines 492 - 498):**

*“Notably, the SHAP dependence plot further reveals a complex non-linear association between the GCS score and SAD. Clinically, GCS scores in the 1-3 range typically indicate severe brain injury, while scores of 14-15 signify a state of clear consciousness. Interestingly, the incidence of delirium in both these extreme scoring groups is significantly lower than in patients with intermediate scores. This phenomenon is consistent with findings from multiple prior delirium prediction models and has been reported in the literature.”*

Fig 6(A - C)

8. Please address lack of external validation.

**Reply:** We greatly appreciate your high attention to the model's generalization ability. We fully recognize the importance of independent external validation for the clinical value of predictive models. We have constructed an external validation cohort using the authoritative public database MIMIC-III (AUC=0.70). Notably, although both belong to the MIMIC series, MIMIC-IV and MIMIC-III are essentially independent databases. We strictly ensured zero patient overlap between the two databases, with a data collection interval of over a decade and significant generational differences in medical technology—thereby fully ensuring the complete independence of MIMIC-III as an external validation set. Baseline characteristics of the external validation cohort data are detailed in S3 Table.

**Revised Text (lines 312 - 332):**

*“Upon identifying 17 clinical features, we employed eight distinct machine learning algorithms to construct predictive models for SAD. Following the application of the SMOTE function to balance the training set, the models developed on internal and external validation sets—specifically LR, SVM, GBM, Neural Network, RF, XGBoost, AdaBoost, and LightGBM—achieved AUC values of 0.67, 0.68, 0.73, 0.69, 0.71, 0.68, 0.69, and 0.70, respectively (Fig 4A). Corresponding AUC values for the external validation set were 0.61, 0.61, 0.70, 0.72, 0.70, 0.65, 0.66, and 0.67 (Fig 4B). Detailed performance metrics, including Accuracy, Sensitivity, Specificity, Precision, and F1-Score for both internal and external validation sets, are provided in Table 3. Due to the substantial performance gap observed between the training and validation sets for the RF model, raising concerns about potential overfitting, the GBM model was ultimately selected as optimal. To further evaluate the GBM model's performance, we generated calibration curves and Decision Curve Analysis (DCA) curves for both the internal and external validation sets (Fig 4C - F). The GBM model's calibration curve and DCA demonstrated robust predictive performance across the majority of threshold probability ranges and indicated significant clinical net benefit. Based on the comprehensive assessment of the above performance metrics, the GBM model was confirmed as the most suitable predictive tool for this dataset and was selected for subsequent analyses. A confusion matrix illustrating the GBM model's classification performance for patients in the internal validation set is presented (Fig 4E). Finally, the feature importance plot for the GBM model is also shown (Fig 4F).”*

9. Discuss missing biomarkers (PCT/IL-6) as future enhancements.

**Reply:** Thank you for your insightful comment. We agree that inflammatory biomarkers such as procalcitonin (PCT) and interleukin-6 (IL-6) are highly relevant to the pathophysiological mechanisms of sepsis and delirium. However, these variables were not included in the current study due to their limited availability and high proportion of missing data in the MIMIC-IV database. We have now addressed this point in the revised Limitations section of the manuscript **(lines546-555)**, and have emphasized that the absence of these biomarkers may have limited the model’s ability to capture neuroinflammatory aspects of SAD. We also added a statement outlining our plan to incorporate these biomarkers in future prospective, multi-center studies to further enhance the model’s predictive performance and biological interpretability.

**Revised Text (lines 312 - 332):**

*“Additionally, this study did not include key inflammatory biomarkers such as procalcitonin (PCT) and interleukin-6 (IL-6), which are closely associated with the pathophysiology of sepsis and delirium. However, due to their limited availability and high rates of missingness in the MIMIC-IV database, these variables could not be incorporated into the current model. This omission may have constrained the model’s ability to capture certain inflammatory dimensions of SAD. In future work, we plan to conduct prospective, multi-center studies that systematically collect longitudinal data on biomarkers such as PCT and IL-6. Integrating these markers may improve the predictive performance and biological interpretability of the model, thereby enhancing its clinical utility and generalizability.”*

10. Table 3: Correct AdaBoost metrics (validation accuracy seems implausible).

**Reply:** We deeply appreciate the reviewer's meticulous review and for highlighting the concerns about the AdaBoost model metrics in Table 3 of the validation set. Upon thorough investigation, we identified that the issues with the original validation accuracy primarily stemmed from the class imbalance between SAD-positive and SAD-negative cases in the training data. This imbalance likely caused the AdaBoost model to exhibit bias toward the majority class during training, potentially leading to skewed evaluation results on the original validation set. To address this, we rigorously reapplied the SMOTE to the training set, expanding the sample size to achieve balance: 7,065 SAD cases and 7,065 non-SAD cases. The model was then retrained using this balanced dataset and reevaluated on the original validation set. This process enabled a thorough revision of the AdaBoost validation metrics. The performance indicators in Table 3 have now been comprehensively updated based on this rigorous recalibration. These revised results provide a more accurate and reliable representation of the optimized AdaBoost model’s capability in identifying SAD risk. The methodology and rationale for these corrections have been explicitly documented in the revised manuscript.

Table 3 **The predictive capabilities of each model.**

| **Model** | **Accuracy** | **Sensitivity** | **Specificity** | **Precision** | **F1-Score** |
| --- | --- | --- | --- | --- | --- |
| Internal validation sets |  |  |  |  |  |
| Logistic | 0.536 | 0.747 | 0.48 | 0.275 | 0.402 |
| SVM | 0.586 | 0.73 | 0.548 | 0.299 | 0.424 |
| GBM | 0.726 | 0.533 | 0.777 | 0.386 | 0.448 |
| NeuralNetwork | 0.606 | 0.703 | 0.581 | 0.307 | 0.427 |
| RandomForest | 0.714 | 0.509 | 0.768 | 0.367 | 0.426 |
| Xgboost | 0.675 | 0.549 | 0.708 | 0.331 | 0.413 |
| Adaboost | 0.491 | 0.862 | 0.393 | 0.273 | 0.414 |
| LightGBM | 0.68 | 0.558 | 0.712 | 0.338 | 0.421 |
| External validation sets |  |  |  |  |  |
| Logistic | 0.572 | 0.692 | 0.438 | 0.579 | 0.63 |
| SVM | 0.578 | 0.646 | 0.501 | 0.591 | 0.618 |
| GBM | 0.578 | 0.318 | 0.869 | 0.731 | 0.443 |
| NeuralNetwork | 0.664 | 0.645 | 0.684 | 0.695 | 0.669 |
| RandomForest | 0.586 | 0.342 | 0.859 | 0.73 | 0.466 |
| Xgboost | 0.554 | 0.308 | 0.828 | 0.667 | 0.421 |
| Adaboost | 0.627 | 0.791 | 0.443 | 0.614 | 0.691 |
| LightGBM | 0.586 | 0.381 | 0.814 | 0.696 | 0.493 |

11. Abbreviations: Define all acronyms at first use (e.g., SOFA, GCS in Abstract).

**Reply:** Thank you for your helpful suggestion. We have revised the abstract to ensure that all abbreviations, including Sequential Organ Failure Assessment (SOFA) and Glasgow Coma Scale (GCS), are defined at their first mention. Additionally, we have thoroughly reviewed the entire manuscript and ensured that all acronyms are consistently defined upon first use throughout the text, in accordance with journal guidelines.

**Revised Text (lines 44 - 46):**

*“The SHAP feature importance ranking revealed five critical risk factors for predicting outcomes: Glasgow Coma Scale (GCS), ICU stay duration, chloride, sodium, and Sequential Organ Failure Assessment (SOFA).”*

12. References: Format consistently (e.g., Ref 38 lacks commas between authors).

**Reply:** Thank you for pointing this out. We have carefully reviewed and revised the reference list to ensure consistency in formatting. Specifically, we corrected Reference 38 by inserting the missing commas between author names. We also re-checked all references to ensure they conform to the journal’s citation style requirements.

**Revised Text (lines 669):**

*“40.	Gong K, Lee HK, Yu K, Xie X, Li J. A prediction and interpretation framework of acute kidney injury in critical care. J Biomed Inform. 2021;113:103653.”*
